# RACIAL EQUITY IN NEW DEAL EMPLOYMENT AND LONG-TERM MORTALITY IN US COUNTIES

**DOI:** 10.1093/geroni/igae098.1750

**Published:** 2024-12-31

**Authors:** David Rehkopf, Ivan Mejia Guevara, Sepideh Modrek

**Affiliations:** Stanford University, Palo Alto, California, United States; Stanford University, Stanford, California, United States; San Francisco State Univeresity, San Francisco, California, United States

## Abstract

Public Emergency Work was a critical policy action during the New Deal used to address the unemployment of the Great Depression (1929-1941). Due to local control of hiring, there were substantial geographic differences in Public Emergency employment by race, relative to need for work. We examined the associations of Black-White differences in the relative equity of county level Public Emergency Work relief in 1940 with county level age 65 to 84 mortality from 2000 to 2019. We find that counties that strongly favored Black workers in Public Emergency Work programs in 1940 had lower mortality rates for both Black and White individuals from 2000 to 2019, as compared to counties that strongly favored hiring White workers. These findings suggest that there are meaningful connections between the structures that influenced the local level implementation of social policies at least as far back as 1940, with recent county mortality differences.

